# Editorial Review for: Why Is There Low Utilization of Biosimilars in Inflammatory Bowel Disease Patients by Gastroenterology Advanced Practice Providers?

**DOI:** 10.1093/crocol/otab005

**Published:** 2021-04-02

**Authors:** Karen Hanson

**Affiliations:** Associate Editor for Crohn’s & Colitis 360 Journal

The authors of this article surveyed Gastroenterology advanced practice providers (APPs) at 2 national conferences regarding their practice patterns and associated attitudes in prescribing biosimilars to patients with inflammatory bowel disease (IBD).

To briefly review the background the first biosimilar to infliximab was approved for use in the United States in 2016. Biosimilarity is somewhat of a new concept that means having sufficient similarity of molecular structure, biological activity, efficacy, safety, and immunogenicity to the originator biologic.^1^ An important distinction is that a biosimilar is not considered a generic because they are synthesized from living cells and cannot be equivalent.^2^ The approval process for biosimilars is less extensive than it is for originator biologics and places more emphasis on comparative analytical data than on clinical studies.^1^ Though biosimilars have been approved and adopted widely in Europe, the authors note there are currently 2 (of 4) FDA approved biosimilars to infliximab that are on the market in the United States (infliximab-dyyb and infliximab-abda). There are 4 biosimilars to adalimumab that are approved by FDA but are not yet on the market although expected to be available in 2023.^3^

Biologics are very effective for inducing and maintaining remission in IBD, but because of the cost access can be a problem. The major driver for using or switching to a biosimilar is the cost savings to insurance payors, the health care system and potentially to the patient. As the authors note, interchangeability and similarity in safety and efficacy have been demonstrated between the originator biologic and an FDA approved biosimilar. These factors suggest they are acceptable alternatives in clinical practice.

The authors identified APP knowledge deficits regarding biosimilar safety and efficacy. Most of those surveyed were not comfortable and not likely to start prescribing within the next year. This survey begs further exploration of what factors may be at play here and also highlights the need to understand barriers that APPs face as key frontline providers on the IBD care team. The lack of confidence in prescribing biosimilars is complex and likely related to a several systemic issues:

1. Education and training play a role in APP confidence. In the United States, there are many APPs in specialty and subspecialty practices. In IBD they evaluate, initiate treatment, and manage patients with complex disabling diseases, often independently. To provide context here, APP formal schooling is primary care based. A review of the literature revealed an older 2010 article detailing results of an internet survey of APPs working in IBD practices.^4^ This survey showed that APPs were spending 86% of their time in autonomous (independent) patient care practices. Only 7% were in academic settings while 92% were in community clinics. Most (63%) did not receive formal training to specialize in complex IBD care other than what was offered on the job.^4^ To reiterate, this survey was conducted 10 years ago and there are certainly more emerging educational resources in the United States today. Yet it is unclear whether the educational opportunities are readily accessible in all settings. The critical need to educate APPs who function as IBD specialists is becoming more and more recognized by groups such as American Gastroenterology Association (AGA University), Crohn’s & Colitis Foundation (CCF), and American College of Gastroenterology (ACG), and Gastroenterology & Hepatology Advanced Practice Providers (GHAPP) all of whom are expanding education offerings in various formats such as preceptorships, online university webinars, lunch and learn sessions, and national conferences to meet the needs of APPs when caring for complex IBD patients.

In terms of biosimilar education, an interesting study from the United Kingdom looked at implementing a nurse led education program and structured communication strategy when switching to a biosimilar.^5^ In this study, patients were approached by a clinical nurse specialist while receiving their originator biologic infusion, and given an information sheet on biosimilars. They were subsequently given the opportunity to agree to this change after discussing with the APP and then followed over time to evaluate for any side effects or loss of response. The study demonstrated a reduction in cost while preserving safety and efficacy in the 143 patients who agreed to participate. Additionally, patient confidence and adherence rates improved.^5^ This proactive approach of introducing the biosimilar in a methodical way to providers and patients could potentially allow for buy in from all members of the team. Additionally, professional groups such as GHAPP, ACG, AGA, and CCF that provide educational offerings for APPs could potentially be explored as a trusted venue for biosimilar information and updates.

1. APPs practice independently, but also team with their physician colleagues in specialty care and will typically align with the practice patterns in their setting. If biosimilar therapies are adopted by their physician teams and/or the workplace setting that can also impact the comfort level of the individual APP to prescribe. Yet, in a survey of 105 physician specialists in 2019, physicians reported that their confidence in using a biosimilar was the biggest barrier to prescribing.^3^ They identified their first priority as the reduction in cost to the patient with a second priority of reduction of cost to the health care system. Fifty-one percent felt knowledgeable and confident in using a biosimilar but this decreased significantly when asked to switch a patient who was actively on treatment and doing well with an originator biologic.^3^
2. The process of how biosimilars are introduced in the IBD setting is a factor and at least initially, the introduction meant a switch from an originator biologic. The insurance payor or health care plan would initiate the change with the response from providers and patients being more reactive than proactive. When introduced by the insurance payer, patients tend to raise concerns to their provider. APPs, as ardent patient advocates, respond cautiously when it is the recommendation of the insurance payer to switch to a biosimilar in part due to their patient’s concerns but also to lack of involvement in the decision to switch. For those patients who have been on a long journey to remission on an effective biologic it can be difficult to make a convincing argument to switch especially when knowledge and confidence are lacking.
3. The concept of interchangeability is new in the pharmaceutical world, so even the language of biosimilars is not well defined. A comparison of brand vs generic medication could be made to illustrate the cost saving potential but as previously stated this is not accurate because they are not equivalent.
4. It is still early. There are only 2 biosimilars to infliximab in use since 2016 in the United States and there was some lag time before they were widely available. Today the originator infliximab biologic still maintains about 85%–90% of the market share, so we are not seeing biosimilars in practice on a regular basis yet.^6^ Exposure will only increase in time especially when adalimumab biosimilars are projected to enter IBD practices in the United States in a few years. That exposure should lead to more confidence in the safety, efficacy, and interchangeability of biosimilars. It would be interesting to repeat this type of survey in a few years.

The authors have started the conversation about a timely and important topic. APPs are uniquely positioned to play a role in the adoption of biosimilars as they become more and more prevalent in our practices with time. We all need to be mindful of the potential for cost saving advantage of biosimilars while preserving safety and efficacy which is beneficial to not only payers but the health care system and to our patients.
